# Special issue European Journal of Physiology: Artificial intelligence in the field of physiology and medicine

**DOI:** 10.1007/s00424-025-03071-x

**Published:** 2025-03-11

**Authors:** Anika Westphal, Ralf Mrowka

**Affiliations:** 1 Thüringer Innovationszentrum Für Medizintechniklösungen (Thimedop), Universitätsklinikum Jena, Jena, Germany; 2 Experimentelle Nephrologie, KIMIII, Universitätsklinikum Jena, Friedrich-Schiller-Universität Jena, Jena, Germany

**Keywords:** Artificial intelligence, Explainable AI, Ethics of AI, Histopathology

## Abstract

This special issue presents a collection of reviews on the recent advancements and applications of artificial intelligence (AI) in medicine and physiology. The topics covered include digital histopathology, generative AI, explainable AI (XAI), and ethical considerations in AI development and implementation. The reviews highlight the potential of AI to transform medical diagnostics, personalized medicine, and clinical decision making, while also addressing challenges such as data quality, interpretability, and trustworthiness. The contributions demonstrate the growing importance of AI in physiological research and medicine, the need for multi-level ethics approaches in AI development, and the potential benefits of generative AI in medical applications. Overall, this special issue showcases some of the the pioneering aspects of AI in medicine and physiology, covering technical, applicative, and ethical viewpoints, and underlines the remarkable impact of AI on these fields.

Artificial intelligence (AI) has witnessed tremendous growth and advancements in recent years and has transformed many aspects of our lives. From virtual assistants to self-driving cars, AI-assisted diagnostic procedures in medicine, health care administration, and AI-powered decision making systems [5] may increasingly become an integral part of our daily experiences. In this special issue, we brought together recent topics related to artificial intelligence in connection with physiology and medicine, focusing on specific technical aspects and applications as well as ethical issues.

Histopathology is an important method in medical diagnostics because it allows for the microscopic examination of tissue samples, enabling clinicians to identify abnormalities and make image-based diagnoses of various diseases, including cancer. In the classical approach, a well-trained pathologist makes the diagnosis by inspecting the microscopic images, and based on experience and published criteria, a diagnosis or classification is made. In a systematic review, Hölscher et al. discuss the current status of the application of digital histopathology [4]. The authors discuss the evolution of histopathology from manual analysis to digital pathology and computational pathology, which can be used in the context of artificial intelligence and deep learning techniques to analyze pathology specimens. The review of Hölscher et al. also highlights the trend towards using foundation models in computational pathology, which are pre-trained on a wide range of data and can be adapted for various tasks. Examples of such models include Virchow, UNI, and CONCH, which have shown impressive results in cancer detection, tumor lymphocyte detection, and other tasks. The authors conclude that histopathology has undergone a significant transformation from manual analysis to digital and computational pathology, which uses AI and deep learning techniques to analyze specimens with high precision and accuracy, enabling automated classification, segmentation, and regression, and ultimately advancing towards precision medicine and understanding disease mechanisms. While there have been significant advances in this field, there are still hurdles to overcome before clinical implementation, including limited digitalization of pathology institutes, lack of prospective evidence, and reimbursement challenges.

Perhaps the most known example of a generative AI is the large language model ChatGPT of OpenAI [8]. Generative AI may create text or image data [6] based on training in huge amounts of data. An illustrative example of an image generated by a generative AI is shown in Fig. 1. The potential of *generative AI* in physiology is manifold. Umesh et al. highlight the motivations and opportunities of generative AI as well as the challenges of analyzing tabular patient data [9]. They describe opportunities in data augmentation and data collaboration, for example, in matching in vivo and in vitro data of studies and for decision making in the clinical context, e.g., for the selection of suitable cancer therapy and in personalized medicine. They describe the technical aspects of state-of-art models, e.g., generative adversarial networks (GANs) and diffusion models and common evaluation measures available. Physiological complexity, linked tables, and diverse feature types are fundamental challenges of tabular data generative approaches. Clinical research, personalized medicine as well as healthcare policy can benefit from data created by generative AI. Umesh et al. highlight the promising benefits of generative AI in clinical trial processes, in drug discovery, digital twins, and data enrichment, as well as data sharing techniques.

**Fig. 1:**
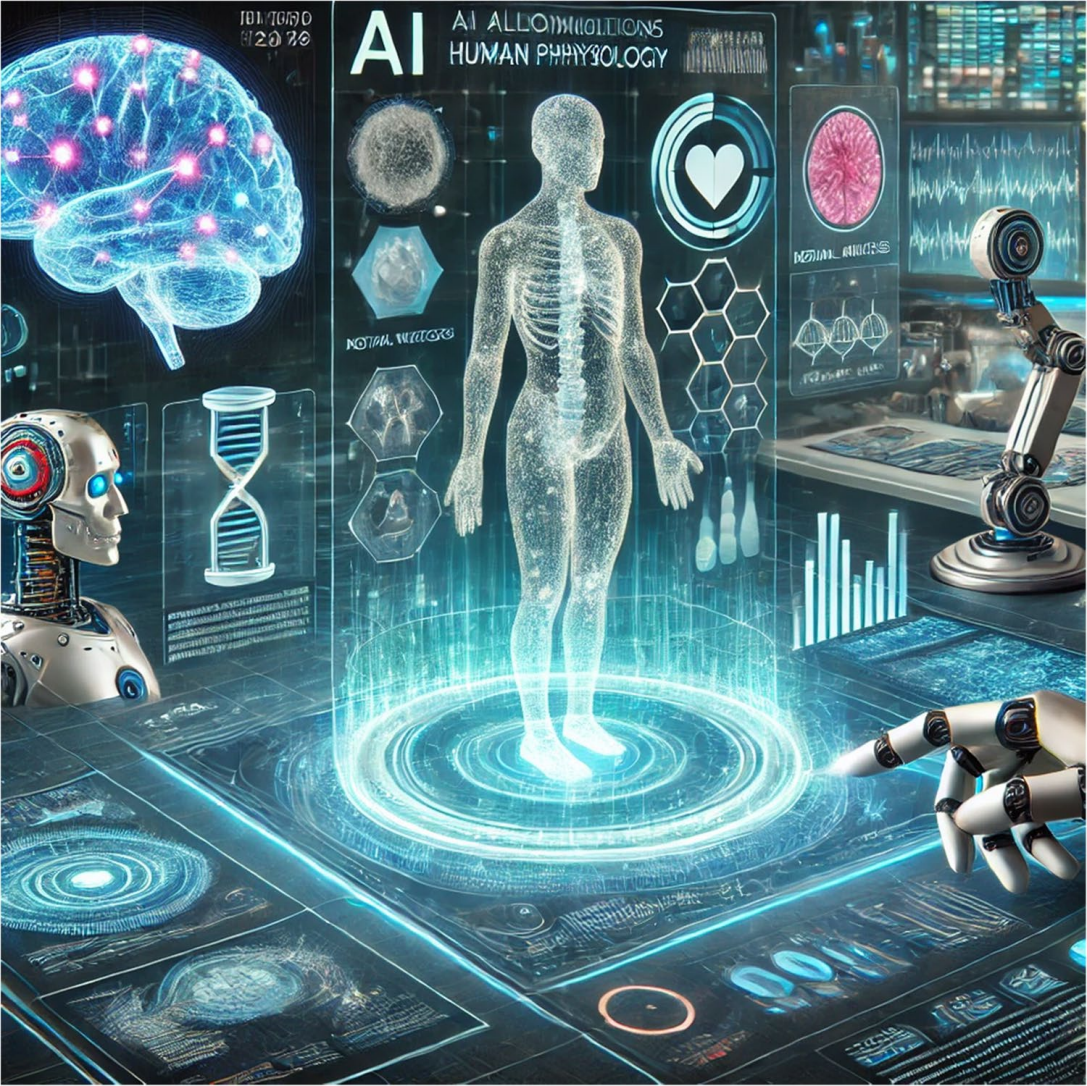
Example of an AI-generated image (ChatGPT, 14 Feb 2025) for the topic: AI algorithms in physiology

The current state of the art in generative modeling and its enormous potential in neurological diseases research is the topic of the review by Seiler and Ritter [7]. They describe various model types of (deep) generative modeling, e.g., variational autoencoder and normalizing flows as well as different goals for its usage in neuroimaging, especially new data generation and representation learning. Current clinical applications of generative modeling in terms of Alzheimer’s disease and multiple sclerosis are described in detail, and the authors present challenges and concerns for data resources, evaluation, and privacy.

Explainable Artificial Intelligence (XAI) is a field of study focused on developing machine learning tools that provide transparent and understandable explanations for their predictions or decisions. By bridging the gap between complex AI algorithms and human users, XAI is an important approach to enhancing trust, accountability, and comprehension. XAI in general potentially offers insights into how inputs are transformed into outputs through several visualization techniques such as global explanations (understanding overall decision-making) and local explanations (identifying key input features influencing specific decisions). In this special issue, we have three contributions in that direction:

The review by Finzel [3] focuses on the novel field of *explainable artificial intelligence* in a more general view. XAI aims to make artificial intelligence comprehensible and explainable, in the sense of verification. Finzel gives short introductions to XAI methodologies as well as current publications in the field of XAI—providing the method, research focus, and medical background. Finzel discusses the growing importance of Explainable Artificial Intelligence (XAI) in physiological research, where AI is increasingly used for analytical and predictive purposes. The review of Finzel is based on 85 papers, she reviewed key topics in XAI, provided an overview of current methods applied in physiology, and discussed solved and unresolved challenges using practical medical examples. It also explores two future directions: firstly, developing trustworthy AI for integrative physiological research and secondly, integrating physiological expertise into XAI development to foster effective human-AI partnerships. The review by Finzel concludes that Explainable Artificial Intelligence has significant potential to enhance physiological research by enabling knowledge discovery and integrating human expertise with models and data. Finzel highlights that physiology could greatly benefit from XAI methods, while XAI itself could improve by incorporating physiological insights to generate more meaningful explanations. The ultimate goal is to create human-centered explanations for AI decisions, which would foster a more seamless and integrative use of AI in physiology and medicine.

Boge and Mosig [1] focus on how biomedical decisions of artificial intelligence can be explained and declared as trustworthy, especially in medical diagnostics. The authors explain the necessity of explainability using an example from the seafaring era and depict recent cases in pathology that are based on AI, e.g., for colorectal cancer. They give a comprehensive overview about the main aspects of explainability, the relationship between causality and explanation, and robustness and trustworthiness in terms of AI in biomedical approaches. Finally, the authors provide several guidelines for connecting scientific explanations and AI in biomedicine. In addition, the authors highlight that another area left for future work is determining what makes a hypothesis a strong and useful explanation in specific medical applications, as well as how much explanation a particular AI system requires. The authors suggest that this will need to be evaluated on a case-by-case basis. They also note that while some studies identify explainable explanations in principle, systematic validation of these explanations has not yet been conducted in practice. This further illustrates the potential of scientific explanation through an example in computational pathology, where it distinguishes microsatellite-stable from microsatellite-unstable tumors in colorectal tissue, weighing the costs and benefits of such an approach. The authors project that trustworthy AI systems will require tailored explanations, especially when unexplained failures occur, arguing that approaches to scientific explanation provide a pathway to achieving this goal.

In a more specific systematic review, Contreras et al. [2] explore the current state of XAI in spectroscopy, a relatively new area. After analyzing 21 studies from a search of major journal databases, the authors find that most research focuses on using XAI methods for spectral data analysis to identify significant spectral bands. The most commonly used AI techniques are SHAP, LIME-inspired masking methods, and CAM, which offer interpretable explanations without modifying original models. The authors conclude in that review that future research should develop new methods and adapt XAI approaches from other domains to better suit spectroscopic data’s unique characteristics [2].

With all types of revolutions in history, there are aspects of ethical concerns to be considered. In that regard, we have included a contribution with an ethical focus on AI. Vandemeulebroucke [10] claims that artificial intelligence systems in healthcare and medicine promise improved care quality, efficiency, and cost reduction. However, he also convincingly describes persisting concerns regarding privacy, power dynamics, and biases. While existing ethical approaches provide a framework, they often narrowly focus on specific settings, neglecting broader global and environmental impacts. To address this, he proposes a multi-level ethics approach—spanning individual, organizational, societal, global, and historical levels—to ensure that AI systems meet universal needs responsibly.

To conclude, in this special issue, we present a review collection that deals with many pioneering aspects of artificial intelligence in medicine and physiology, from technical, applicative, and ethical viewpoints. Just as AI is revolutionizing the lives of many, the impact in medicine and physiology is also remarkable. We thank all authors for their valuable contributions.

## Data Availability

No datasets were generated or analysed during the current study.
